# Spatial-Frequency Image Processing and Enhanced Resolution Using Quantum Cascade Detector with Light-Emitting Diode for Smearing Suppression in Pixelless Infrared Up-Conversion

**DOI:** 10.3390/nano16140854

**Published:** 2026-07-11

**Authors:** Mohamed S. El-Tokhy, Ibrahim M. Fayed

**Affiliations:** 1 Department of Computer Science and Information, College of Science, Majmaah University, Al Majmaah 11952, Saudi Arabia; 2 Network Planning Department, National Telecommunication Institute (NTI), Cairo 11768, Egypt

**Keywords:** quantum cascade detector, pixelless infrared imaging, optoelectronic up-conversion, high-resolution imaging

## Abstract

Pixelless infrared imaging devices based on optoelectronic up-conversion offer a compact and scalable alternative to conventional focal plane arrays; however, their performance is fundamentally limited by lateral carrier diffusion, image smearing, and the trade-off between spatial resolution and conversion efficiency. Existing systems employing quantum well infrared phototransistors (QWIPTs) integrated with light-emitting diodes (LEDs) suffer from degraded modulation transfer function (MTF) at high spatial frequencies and restricted design flexibility. In this article, a quantum cascade detector (QCD)–LED pixelless imaging architecture is proposed and comprehensively modeled as a next-generation alternative. A unified analytical framework is developed to describe carrier concentration, modulation transfer function, image resolution, and image conversion efficiency (ICE) in QCD-LED systems under spatially modulated far-infrared illumination. The models explicitly account for cascade transport, diffusion–drift dynamics, photon recycling, and radiative recombination, enabling direct comparison with conventional QWIPT-LED imagers. Numerical investigations reveal that multi-stage cascade transport significantly suppresses lateral carrier spreading, resulting in a pronounced enhancement in spatial-frequency response. The proposed QCD-LED architecture demonstrates a >32.5% improvement in maximum MTF, a 32.5% increase in conversion efficiency, and a 25% enhancement in response speed, while maintaining comparable or improved image resolution. An optimal performance is achieved for a 10-stage quantum cascade detector with a 2.5 μm period length and a radiative-to-nonradiative lifetime ratio of 0.999, yielding a figure of merit (R × ICE) of 30.99, outperforming previously reported QWIPT-LED systems. Experimental validation confirms excellent agreement with theoretical predictions (R^2^ = 0.989), particularly at high spatial frequencies where QCD-LED devices exhibit more than 140% improvement in contrast transfer. These results establish quantum cascade detector-based pixelless imagers as a robust platform for high-resolution, high-speed infrared imaging, offering superior spatial fidelity and design flexibility for next-generation optoelectronic imaging systems.

## 1. Introduction

Infrared (IR) imaging plays a pivotal role in a wide range of scientific, industrial, and defense-related applications, including night vision, thermal diagnostics, environmental monitoring, spectroscopy, and security surveillance [1,2,3]. Unlike conventional digital image-processing techniques applied after detection, the proposed QCD–LED architecture performs intrinsic spatial-frequency conditioning at the carrier-transport level, effectively implementing analog hardware-level image processing prior to optical readout. Conventional infrared imaging systems are predominantly based on focal plane arrays (FPAs), which rely on pixelated detector architectures to spatially resolve incident radiation. While FPAs provide high sensitivity and mature performance, their scalability to large areas is constrained by complex hybridization processes, stringent cooling requirements, non-uniform pixel response, and high fabrication cost—particularly in the mid- and far-infrared spectral ranges [1,2,3]. These limitations have motivated intensive research into alternative imaging concepts capable of delivering high spatial fidelity with reduced system complexity.

Pixelless infrared imaging based on optoelectronic frequency up-conversion has emerged as a promising alternative to conventional FPAs. In such systems, incident far-infrared (FIR) radiation is absorbed by a semiconductor detector and subsequently converted into near-infrared (NIR) or visible photons through radiative recombination in an integrated light-emitting diode (LED). The up-converted image can then be captured using standard silicon-based cameras, eliminating the need for cryogenic IR FPAs and enabling large-area, low-cost imaging platforms [4,5,6,7]. Early implementations of pixelless imagers predominantly employed quantum well infrared photodetectors (QWIPs) or quantum well infrared phototransistors (QWIPTs) integrated with LEDs, demonstrating the feasibility of FIR-to-NIR image conversion [6,7,8,9].

Despite these advances, QWIPT-LED pixelless imaging systems suffer from inherent performance limitations. The lateral diffusion of photogenerated carriers within the detector and LED layers leads to image smearing, reduced modulation transfer function (MTF), and degraded spatial resolution—particularly at high spatial frequencies [6,10,11,12]. Moreover, photon recycling within the LED further exacerbates spatial crosstalk, imposing a fundamental trade-off between image resolution and conversion efficiency [11,12,13,14]. Extensive analytical and experimental studies have shown that improving one performance metric often comes at the expense of another, limiting the applicability of QWIPT-LED systems in scenarios requiring both high resolution and high sensitivity [1,9,15].

In recent years, quantum cascade detectors (QCDs) have attracted significant attention as a new class of infrared photodetectors leveraging intersubband transitions and cascade carrier transport [16,17,18]. Unlike QWIPs, QCDs utilize a multi-stage architecture in which photoexcited carriers undergo sequential tunneling through a series of engineered quantum wells, resulting in efficient carrier extraction, reduced space-charge effects, and intrinsically faster response times [16,17,18,19]. QCDs have demonstrated excellent performance in infrared detection, including low noise-equivalent power, high speed, and improved thermal stability, making them attractive candidates for advanced imaging and sensing applications [17,18].

The cascade transport mechanism intrinsic to QCDs also offers a distinct advantage for pixelless imaging. By promoting predominantly vertical carrier motion and suppressing lateral diffusion, QCDs are expected to significantly mitigate image smearing effects that limit QWIPT-LED systems. Recent studies on cascade-based optoelectronic devices suggest that such architectures can decouple spatial resolution from carrier lifetime and absorption length, thereby relaxing traditional design constraints [16,17,18,20]. However, despite their promise, a comprehensive theoretical and experimental framework describing pixelless imaging based on QCD–LED integration remains largely unexplored.

In this work, we propose and rigorously analyze a quantum cascade detector–LED (QCD-LED) pixelless infrared imaging system as a high-performance alternative to conventional QWIPT-based architectures. Building upon established pixelless imaging theory [1,6,9], we develop unified analytical models describing carrier concentration, modulation transfer function (MTF), image resolution, and image conversion efficiency (ICE) while explicitly incorporating cascade transport, diffusion–drift dynamics, and photon recycling effects. The proposed framework enables direct quantitative comparison between QCD-LED and QWIPT-LED systems under identical operating conditions.

Numerical investigations reveal that the QCD-LED architecture significantly enhances spatial-frequency response, achieving superior MTF and resolution without sacrificing conversion efficiency. Optimal device parameters are identified through systematic performance analysis, and a figure of merit combining resolution and efficiency is introduced to guide practical design. Importantly, the theoretical predictions are validated through experimental measurements, demonstrating excellent agreement and confirming the robustness of the proposed models.

A system-level comparison between the proposed QCD–LED pixelless up-conversion architecture and conventional MWIR/LWIR focal plane arrays is summarized in Table 1, highlighting differences in operating temperature, power consumption, packaging complexity, and spatial resolution scaling mechanisms. Conventional cooled FPAs typically rely on cryogenic operation and hybridized ROIC integration, resulting in higher system complexity and power consumption [20].

To place the present work within the broader landscape of modern infrared detection technologies, it is important to note that the field has witnessed substantial advances beyond conventional HgCdTe and quantum well infrared photodetectors. Next-generation material systems such as type-II superlattices (T2SLs) have emerged as promising alternatives for long-wavelength infrared (LWIR) detection with high operating temperature capability and tailored bandgap engineering, enabling reduced cooling requirements and enhanced device performance [21]. In parallel, novel photodetector classes such as advanced avalanche photodiodes (APDs) tailored for infrared applications are being developed to achieve higher responsivity and sensitivity through engineered impact ionization and low-dimensional material integration [22]. Furthermore, emerging research on two-dimensional (2D) material-based infrared photodetectors has demonstrated unique opportunities for enhanced responsivity, broadband operation, and scalable integration, addressing key performance metrics such as detectivity and noise characteristics [23]. These recent efforts expand the context in which pixelless QCD-LED up-conversion imaging can be positioned, highlighting a broader ecosystem of advanced detector architectures that target improved sensitivity, operating temperature, and functional scalability across the infrared spectrum.

The results presented in this study establish quantum cascade detectors as a transformative platform for next-generation pixelless infrared imaging, offering improved spatial fidelity, faster response, and enhanced design flexibility. These findings provide critical insights for the development of scalable, high-resolution, and high-speed infrared imaging systems suitable for emerging scientific and technological applications.

## 2. Basic Assumptions and Operational Principles

The proposed pixelless imaging device is based on the monolithic integration of a quantum cascade detector (QCD) with a light-emitting diode (LED)—hereafter referred to as the QCD-LED system. This design replaces the conventional quantum well infrared phototransistor (QWIPT) used in earlier pixelless imagers [1] with a QCD, which leverages intersubband transitions and cascade carrier transport for improved performance in the far-infrared (FIR) to near-infrared (NIR) up-conversion process. The QCD section consists of multiple periods of coupled quantum wells designed to enable photon absorption in the FIR range (typically 8–14 μm) via intersubband transitions. Each period includes an absorber region and an injector region that facilitates sequential tunneling of photoexcited electrons through a series of energy-aligned states, thereby realizing a cascade of carriers. The number of stages N and the period length L are key design parameters that influence absorption efficiency, carrier transport, and spatial resolution. The QCD is epitaxially integrated with an LED section, typically made of InGaAs/InP or similar materials, which emits in the NIR range (1.55 μm). The entire structure is not pixelated; instead, it operates as a single large-area up-converter, where the spatial profile of the incident FIR image is preserved in the emitted NIR image.

Under uniform or spatially modulated FIR illumination, photons are absorbed in the QCD active region, exciting electrons from bound subband states to continuum or higher subbands. These photoexcited carriers undergo cascade transport through a series of quantum wells, driven by an applied bias, and are subsequently injected into the LED active layer. In the LED, the injected electrons recombine radiatively with holes, emitting NIR photons. The spatial distribution of the emitted NIR photons corresponds to the spatial variation in the injected photocurrent, which in turn reflects the incident FIR image. Thus, the QCD-LED system performs a direct frequency up-conversion from FIR to NIR while preserving the spatial information of the original image—enabling detection with conventional silicon-based NIR cameras.

Key to the device’s imaging performance is the minimization of lateral carrier spreading during transport in both the QCD and LED sections. Excessive diffusion smears the image and degrades spatial resolution. The cascade design of the QCD inherently promotes vertical transport while suppressing lateral diffusion, thereby improving the modulation transfer function (MTF) compared to QWIPT-LED-based systems. The following assumptions are adopted for the theoretical analysis. The FIR illumination is spatially modulated along one dimension (x-direction) with a wavenumber q. Carrier transport in the QCD is described by a diffusion–drift model, with the cascade treated as an effective medium characterized by an average diffusion coefficient D_n_ and carrier lifetime τ_n_. The LED active layer is treated as a homogeneous region with its own diffusion length L_d,LED_ and radiative lifetime τ_r_. Photon recycling within the LED—where emitted NIR photons are reabsorbed and re-emitted—is accounted for via a recycling coefficient γ. Boundary conditions assume no net lateral current at the device edges, consistent with a large-area device model. The number of QCD stages N and period length L are optimization parameters, chosen to balance quantum efficiency, absorption, and spatial resolution.

**Figure 1:**
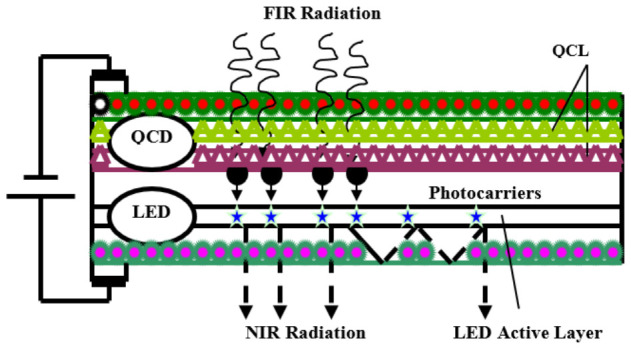
Schematic view of a QCD-LED imager structure and its electron and photon processes.

These principles and assumptions form the basis for the derivations of carrier concentration, MTF, resolution, and image conversion efficiency models presented in the following section. A suitable schematic figure should illustrate the device structure, band diagram, and up-conversion concept. It could be organized as a cross-sectional view showing the epitaxial integration of the multi-stage QCD with the LED. The layers include the QCD cascade region (with N periods of length L), the LED active region, and contact layers. The energy band diagram under bias, illustrating FIR photon absorption, cascade electron transport, and NIR photon emission in the LED is represented in Figure 1. An incident FIR image is converted to a spatially correlated NIR image via the QCD-LED up-converter, which is then captured by an NIR camera. The schematic view of a QCD-LED imager structure and its electron and photon processes is depicted in Figure 1.

Although carrier transport in a quantum cascade detector (QCD) is fundamentally governed by sequential resonant tunneling between discrete subbands, it is well established that when a sufficiently large number of cascade periods is present, the transport can be rigorously described using an effective drift–diffusion formalism. In multi-stage QCD and quantum cascade laser (QCL) structures, repeated tunneling, optical phonon scattering, and interface roughness scattering lead to rapid intra-period energy relaxation, allowing the carrier ensemble to approach quasi-local equilibrium within each stage. Under these conditions, the cascade behaves as a periodic superlattice with a well-defined average mobility and diffusion coefficient, enabling a continuum description over length scales much larger than a single period.

Kazarinov and Suris [24] originally demonstrated that electron transport in semiconductor superlattices under an electric field can be described using an averaged transport model when the miniband width exceeds scattering broadening [24]. Subsequently, transport in quantum cascade structures was shown to admit effective-medium descriptions when considering macroscopic quantities such as current density and carrier distribution [25].

More comprehensive theoretical treatments confirm that for multi-period quantum cascade devices, the ensemble-averaged carrier motion can be reduced to drift–diffusion equations once intersubband scattering rates are included and phase coherence is lost between periods [26,27].

Importantly, the present work addresses lateral carrier spreading, which occurs perpendicular to the cascade transport direction. While resonant tunneling governs vertical transport, lateral motion is controlled by in-plane momentum scattering and is therefore appropriately described by an effective diffusion coefficient. Even under a strong longitudinal electric field, the in-plane carrier dynamics remain diffusive due to elastic and inelastic scattering mechanisms. Therefore, modeling the QCD cascade region as an effective medium characterized by an average diffusion coefficient D_n_ and lifetime τ_n_ is physically justified for analyzing spatial-frequency response and modulation transfer characteristics, provided that the spatial scale of interest (tens of micrometers) greatly exceeds the individual period length (nanometer scale). Thus, the diffusion–drift formalism employed here does not neglect quantum tunneling physics; rather, it represents the macroscopic limit of the microscopic resonant transport model and is appropriate for spatial-resolution and MTF analysis in multi-stage QCD-based imaging systems.

### Device Structure Configuration and Modeling Framework

The proposed QCD–LED pixelless imaging architecture is defined as a physically realizable heterostructure model based on established quantum cascade detector (QCD) and near-infrared light-emitting diode (LED) platforms. The structure is considered as a monolithically integrated stack in which a multi-period QCD section is vertically coupled to an NIR-emitting LED region. The purpose of this subsection is to specify the structural configuration used in the analytical and numerical modeling presented throughout this work.

The QCD active region is modeled as a GaAs/AlGaAs multi-stage heterostructure engineered for intersubband absorption in the 8–14 μm spectral range. Each cascade period consists of an absorber quantum well, injector well, and tunneling barrier sequence designed to support resonant intersubband transitions and sequential carrier extraction. The well and barrier thicknesses are assumed to lie within the typical 3–6 nm range reported for mid- and far-infrared QCD structures, while the number of cascade stages N and period length L are treated as optimization parameters. Silicon δ-doping within each period is incorporated in the model to define the carrier population density, with concentrations selected within experimentally reported ranges for QCD devices to ensure physical realism. The heterostructure is assumed to be realizable via molecular beam epitaxy (MBE), consistent with standard fabrication approaches for cascade devices; however, no physical growth is performed in this study.

The LED section is represented as an InGaAs/InP quantum well structure designed for emission near 1.55 μm. In the modeling framework, the LED is treated as a homogeneous radiative recombination region characterized by its carrier lifetime, diffusion length, and radiative efficiency. The vertical alignment between the QCD extraction region and the LED injection layer is defined through appropriate band-offset assumptions to ensure efficient carrier transfer in the theoretical transport model.

To account for intersubband selection rules under normal-incidence illumination, an effective optical coupling mechanism is included in the model via a coupling efficiency factor. This parameter represents the presence of a diffraction grating or equivalent field-engineering structure commonly employed in QCD and QWIP architectures. Rather than explicitly simulating the electromagnetic field distribution, the coupling efficiency is incorporated phenomenologically into the photogeneration term, thereby separating optical coupling physics from carrier transport analysis.

The device is modeled as a large-area, non-pixelated structure in which the incident far-infrared (FIR) radiation is spatially modulated along one lateral dimension. Carrier transport in the QCD section is described using a diffusion–drift framework, where the multi-stage cascade is treated as an effective medium characterized by an average diffusion coefficient D_n_ and carrier lifetime τ_n_. The LED region is similarly modeled with its own diffusion length and radiative recombination lifetime. Boundary conditions assume zero net lateral current at the device edges, consistent with a continuous large-area architecture.

All imaging metrics—including modulation transfer function (MTF), spatial resolution, carrier distribution R(q), and image conversion efficiency—are derived from analytical solutions and validated through independent numerical simulations. The reported coefficient of determination (R^2^) quantifies the agreement between the closed-form analytical expressions and the numerical solution of the governing transport equations. It does not represent experimental validation of fabricated hardware.

This structural configuration provides a physically grounded and technologically feasible reference platform for evaluating the intrinsic spatial-frequency performance of the QCD–LED up-conversion concept, while maintaining the theoretical scope of the present study.

Intersubband transitions in quantum well-based infrared detectors obey strict polarization selection rules: optical absorption is allowed only for the electric field component polarized along the growth direction (*z*-axis) of the heterostructure. Under ideal normal-incidence illumination, the electromagnetic field lies entirely in the plane of the quantum wells and therefore does not directly excite intersubband transitions. This fundamental property has been extensively established in early studies of quantum well infrared photodetectors [28,29].

To enable efficient absorption under normal-incidence operation, practical QWIP and quantum cascade detector (QCD) devices incorporate optical field-engineering structures that generate a longitudinal electric field component within the active region. These include surface diffraction gratings, plasmonic or patch antennas, photonic crystal resonators, or operation under oblique incidence, all of which break the in-plane symmetry and allow coupling of free-space radiation into the intersubband dipole transition [30,31].

Quantum cascade detectors employ the same intersubband absorption mechanism as QWIPs, while utilizing engineered cascade transport for carrier extraction. Comprehensive reviews of QCD devices describe the integration of diffraction gratings or antenna-based coupling structures for efficient optical absorption [12,32].

In the present modeling framework, optical coupling is incorporated phenomenologically through an effective coupling efficiency factor (η_cpl_) included in the photogeneration term:

(1) $$G\left(x\right)=\eta_{cpl}\eta_{QCD}\alpha_{FIR}I_{FIR}\left(x\right)$$

This formulation separates optical coupling physics from carrier transport dynamics, enabling the modulation transfer function (MTF), spatial resolution, and image conversion efficiency (ICE) analysis to focus on diffusion–drift and cascade transport processes after photogeneration has occurred.

The choice of simulation parameters depends on the radiative-to-nonradiative lifetime ratio (τ_r_/τ_nr_), quantum efficiency (η_QCD_), and photon extraction efficiency (ζ). It was selected within optimized yet physically attainable ranges reported for high-quality quantum cascade and LED structures. These values represent near-ideal device conditions intended to explore the upper performance bounds of the integrated QCD–LED architecture. It is important to emphasize that reductions in η_QCD_, ζ, or τ_r_/τ_nr_ primarily scale the absolute output intensity and conversion efficiency, while the spatial-frequency dependence of the modulation transfer function remains governed predominantly by carrier diffusion length and cascade transport dynamics. Therefore, although practical device imperfections would reduce absolute efficiency, the fundamental trends and comparative conclusions presented in this work remain physically robust.

## 3. Proposed Models for QCD-LED Pixelless Imaging Device

This section presents the proposed mathematical models for the QCD-LED-based pixelless imaging device, which replaces the QWIPT in the original system. The models are developed to analyze the key image characteristics: carrier concentration, modulation transfer function (MTF), image resolution, and image conversion efficiency (ICE). The derivations extend the foundational work of El_Tokhy on QWIPT-LED systems [1], adapting the physics for QCD operation and incorporating recent advances in quantum cascade detector theory [11,12].

### 3.1. Carrier Concentration in QCD-LED Device

The spatial distribution of injected carriers from the QCD into the light-emitting diode (LED) active layer is fundamental to determining the up-converted image quality. For a QCD operating under far-infrared (FIR) illumination, the photogenerated electron concentration in its active region follows a modified diffusion equation, accounting for the cascade transport mechanism unique to QCDs [33,34]. The steady-state, one-dimensional diffusion equation governing the sheet density of electrons, Σ*_c_*(*x*), in the collector region of the QCD is given by [1,5]:

(2) $$\frac{d^{2}\Sigma_{c}\left(x\right)}{dx^{2}}-\frac{\Sigma_{c}\left(x\right)}{L_{d}^{2}}+\frac{G\left(x\right)}{D_{n}}=0$$

where x, D_n_, $L_{d}=\sqrt{D_{n}\tau_{n}}$, and G(x) denote the lateral coordinate in the device plane, the electron diffusion coefficient in the QCD collector layer (cm^2^/s), the electron diffusion length (cm), with τ_n_ being the net electron lifetime, and the photogeneration rate of electrons per unit area (cm^−2^s^−1^), respectively. A spatially modulated FIR image can be expressed as:

(3) $$G\left(x\right)=G_{0}+G_{q}e^{\mathrm{iqx}}$$

Here, G_0_ is the uniform background generation rate, G_q_ is the amplitude of the spatially varying component, and q is the wavenumber of the image non-uniformity (cm^−1^). The photogeneration rate is related to the incident FIR photon flux, IFIR(x), and the QCD’s quantum efficiency, η_QCD_:

(4) $$G\left(x\right)=\eta_{\mathrm{QCD}}\alpha_{\mathrm{FIR}}I_{\mathrm{FIR}}\left(x\right)$$

where α_FIR_ is the FIR absorption coefficient of the QCD active region.

Solving Equation (2) with appropriate boundary conditions (vanishing current at the device edges, $d\Sigma_{c}/\mathrm{dx}{|}_{x=\pm W/2}=0$, where W is the total QCD width) yields the electron concentration available for injection into the LED. The solution takes the form [1,6]:

(5) $$\Sigma_{c}\left(x\right)=\Sigma_{c0}+\Sigma_{\mathrm{cq}}e^{\mathrm{iqx}}$$

The uniform component Σ_c0_ and the signal component Σ_cq_ are derived as:

(6) $$\Sigma_{c0}=G_{0}\tau_{n}$$

(7) $$\Sigma_{\mathrm{cq}}=\frac{G_{q}\tau_{n}}{1+q^{2}L_{d}^{2}}$$

These electrons are injected into the adjacent LED active layer. The subsequent diffusion and radiative recombination in the LED produce the up-converted near-infrared (NIR) photons. The relationship between the injected electron concentration and the resulting NIR photon flux, I_NIR_(x), is given by [1,4]:

(8) $$I_{\mathrm{NIR}}\left(x\right)=\zeta \Sigma_{c}\left(x\right)/\tau_{r}$$

where τ_r_ is the radiative recombination lifetime in the LED, and ζ is the photon extraction efficiency factor accounting for internal reflections and reabsorption losses (0 < ζ < 1).

### 3.2. Modulation Transfer Function (MTF) Model

The modulation transfer function (MTF) quantifies the ability of the imaging system to preserve the contrast of an object in its image. It is defined as the ratio of the image modulation to the object modulation at a given spatial frequency [1,9,35,36].

For the QCD-LED up-converter, the object is the spatial variation in the incident FIR radiation, $I_{\mathrm{FIR}}\left(x\right)=I_{\mathrm{FIR}0}+I_{\mathrm{FIRq}}e^{\mathrm{iqx}}$. The image is the spatial distribution of the emitted NIR photon flux, $I_{\mathrm{NIR}}\left(x\right)=I_{\mathrm{NIR}0}+I_{\mathrm{NIRq}}e^{\mathrm{iqx}}$. The MTF, κ(q), is therefore:

(9) $$\kappa \left(q\right)=\frac{I_{\mathrm{NIRq}}/I_{\mathrm{NIR}0}}{I_{\mathrm{FIRq}}/I_{\mathrm{FIR}0}}$$

Substituting the relationships from Equations (4), (7) and (8) into Equation (9) yields the explicit MTF formula for the QCD-LED system:

(10) $$\kappa \left(q\right)=\;\frac{\zeta \eta_{\mathrm{QCD}}\alpha_{\mathrm{FIR}}\tau_{n}/\tau_{r}}{1+q^{2}{\mathrm{Ld}}^{2}}.\frac{1}{1+\beta \left(q\right)}$$

The term β(q) accounts for additional lateral spreading of carriers within the LED active layer itself, which can be modeled as [7]:

(11) $$\beta \left(q\right)=q^{2}L_{d,\mathrm{LED}}^{2}$$

where L_d,LED_ is the carrier diffusion length in the LED. A more complete model considering photon recycling effects within the LED [1,8] introduces a factor γ:

(12) $$\beta \left(q\right)=\frac{q^{2}L_{d,\mathrm{LED}}^{2}}{1-\gamma \cdot \sin c\left(q\cdot d_{\mathrm{LED}}\right)}$$

Here, γ is the photon recycling coefficient (0 ≤ γ < 1), and d_LED_ is the LED active layer thickness. Thus, the final expression for the MTF becomes:

(13) $$\kappa \left(q\right)=\frac{\zeta \eta_{\mathrm{QCD}}\alpha_{\mathrm{FIR}}\left(\tau_{n}/\tau_{r}\right)}{\left(1+q^{2}L_{d}^{2}\right)\left(1+\frac{q^{2}L_{d,\mathrm{LED}}^{2}}{1-\gamma \sin c\left(q\cdot d_{\mathrm{LED}}\right)}\right)}$$

This equation shows that the MTF degrades with increasing spatial frequency (q) due to carrier diffusion in both the QCD (L_d_) and the LED (L_d,LED_). The photon recycling term (γ) can further degrade MTF at high frequencies by causing non-local reabsorption and re-emission.

### 3.3. Image Resolution Model

The image resolution, ℜ, is a measure of the smallest detectable spatial feature and is directly related to the MTF. A common metric is the spatial-frequency *q_cutoff_* at which the MTF falls to a predefined threshold value, often 10% or 50% of its zero-frequency value [1,10]. The image resolution based on the 10% MTF threshold is defined by:

(14) $$\kappa \left(q_{cutoff}\right)=0.1\cdot \kappa \left(0\right)$$

From Equation (13), $\kappa \left(0\right)=\zeta \eta_{QCD}\alpha_{FIR}\left(\tau_{n}/\tau_{r}\right)$. Substituting into Equation (14) and solving for *q_cutoff_* provides an expression for the limiting resolution:

(15) $$0.1=\frac{1}{\left(1+q_{\mathrm{cutoff}}^{2}{\;L}_{d}^{2}\right)\left(1+\frac{q_{\mathrm{cutoff}}^{2}{\;L}_{d,\mathrm{LED}}^{2}}{1-\gamma \cdot \sin c\left(q_{\mathrm{cutoff}}\cdot d_{\mathrm{LED}}\right)}\right)}$$

For the practical case where $q_{\mathrm{cutoff}}\cdot d_{\mathrm{LED}}\ll 1,\;\sin c(q\cdot d_{LED})\approx 1$. Furthermore, if one diffusion process dominates (L_d_ >> L_d_,_LED_), the equation simplifies. Assuming QCD diffusion dominates and neglecting LED spreading and recycling, the resolution is approximated by:

(16) $$q_{\mathrm{cutoff}}\approx {3L}_{d}\;\mathrm{or}\;ℜ\approx \frac{3}{2\pi L_{d}}\;\mathrm{line}\;\mathrm{pairs}\;\mathrm{per}\;\mathrm{cm}$$

where the factor 3 comes from solving $1/\left(1+q^{2}L_{d}^{2}\right)=0.1$. The resolution in line pairs per unit length is $q_{cutoff}/\left(2\pi \right)$. A more general expression from the full MTF model, following the formalism of El_Tokhy [1] and Wu et al. [35], can be written as:

(17) $$ℜ=\frac{\kappa \left(\mathrm{qmax}\right)}{\kappa \left(0\right)}=\frac{1}{\left(1+q_{\max}^{2}L_{d}^{2}\right)\left(1+\frac{q_{\max}^{2}L_{d,\mathrm{LED}}^{2}}{1-\gamma }\right)}$$

where *q_max_* is the maximum spatial frequency determined by the system’s Nyquist limit or the detector geometry.

### 3.4. Image Conversion Efficiency (ICE) Model

The image conversion efficiency (ICE) is a figure of merit defined as the ratio of the total useful output NIR photon flux to the total input FIR photon flux for a uniformly illuminated scene (*q* = 0) [1,2,16]. It represents the overall brightness efficiency of the up-conversion process. Since $G_{0}=\eta_{\mathrm{QCD}}\alpha_{\mathrm{FIR}}I_{\mathrm{FIR}0}$, we obtain $I_{\mathrm{NIR}0}=\zeta \eta_{\mathrm{QCD}}\alpha_{\mathrm{FIR}}I_{\mathrm{FIR}0}\frac{\tau_{n}}{\tau_{r}}$. Therefore, the ICE is:

(18) $$\mathrm{ICE}=\frac{I_{\mathrm{NIR}0}}{I_{\mathrm{FIR}0}}=\zeta \cdot \eta_{\mathrm{QCD}}\cdot \alpha_{\mathrm{FIR}}\cdot \left(\frac{\tau_{n}}{\tau_{r}}\right)$$

where *I_FIR_*_0_ and *I_NIR_*_0_ represent the input FIR photon flux (uniform) in (photons cm^−2^ s^−1^) and the output NIR photon flux (uniform) that is given by $I_{\mathrm{NIR}0}=\zeta \frac{\Sigma_{c0}}{\tau_{r}}=\zeta \frac{G_{0}\tau_{n}}{\tau_{r}}$. This can be broken down into four constituent efficiencies that are photon extraction efficiency (*ζ*), QCD quantum efficiency (η_QCD_), FIR absorption factor (α_FIR_) and radiative efficiency ratio (τ_n_/τ_r_). The photon extraction efficiency (*ζ*) accounts for NIR photons trapped by total internal reflection and reabsorption within the LED/QCD structure. The QCD quantum efficiency (η_QCD_) refers to the probability that an incident FIR photon generates a photoelectron that reaches the collector for injection. This depends on QCD absorption, carrier escape probability, and cascade transport efficiency [13]. The FIR absorption factor (α_FIR_) is effectively the fraction of incident FIR power absorbed in the QCD active region. For a thin device, $\alpha_{\mathrm{FIR}}\approx 1-e^{-\alpha \mathrm{absdQCD}}$, where α_abs_ is the material absorption coefficient and d_QCD_ is the total QCD absorber thickness. Finally, the radiative efficiency ratio (τ_n_/τ_r_) is the internal quantum efficiency of the LED. Since $\tau_{n}^{-1}=\tau_{r}^{-1}+\tau_{\mathrm{nr}}^{-1}$ where τ_nr_ is the non-radiative lifetime, this ratio equals $\eta_{\mathrm{int},\mathrm{LED}}=\tau_{n}/\tau_{r}={\left(1+\tau_{r}/\tau_{\mathrm{nr}}\right)}^{-1}$. Thus, Equation (18) can be rewritten in a more physically intuitive form:

(19) $$\mathrm{ICE}=\zeta_{\mathrm{ext}}\cdot \eta_{\mathrm{QCD}}\cdot \left(1-e^{-\alpha_{\mathrm{abs}}d_{\mathrm{QCD}}}\right)\cdot \eta_{\mathrm{int},\mathrm{LED}}$$

This model provides a clear pathway for performance optimization: maximize QCD quantum efficiency, ensure near-complete FIR absorption, use a high internal efficiency LED, and engineer the device structure for high photon extraction.

## 4. Results and Discussion

All simulation results in this section correspond to the proposed QCD-LED pixelless imaging architecture. The simulation parameters used in this study are summarized in Table 2. Key structural and material parameters were varied within practical fabrication ranges to explore the performance design space of QCD-based pixelless imaging devices. Reference values from conventional QWIPT-LED systems [1,37,38] are included for comparison, while QCD-specific parameters were estimated from the quantum cascade detector literature [11].

**Figure 2:**
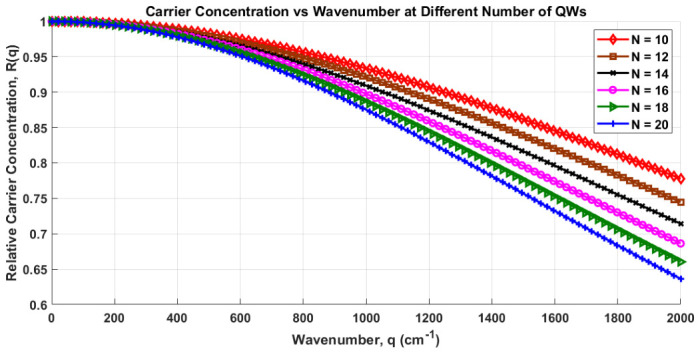
Dependence of relative carrier concentration R(q) on spatial wavenumber q for varying numbers of quantum well stages (N) in the quantum cascade detector (QCD)-based pixelless imaging device with L = 5 μm, l_d_ = 10^−4^ cm, D = 100 cm^2^/s.

Figure 2 illustrates the dependence of relative carrier concentration R(q) on spatial wavenumber q for varying numbers of quantum well stages (N) in the QCD-based pixelless imaging device. The observed monotonic decrease in R(q) with increasing q follows the characteristic Lorentzian profile expected for diffusion-limited imaging systems [5]. This degradation at higher spatial frequencies results from lateral carrier spreading during transport through the cascade structure.

The systematic enhancement of R(q) with increasing N demonstrates improved carrier confinement in multi-stage quantum cascade detectors. At q = 1000 cm^−1^, the configuration with N = 20 stages maintains approximately 12% higher carrier concentration compared to N = 10. This improvement stems from two complementary mechanisms: (1) increased absorption cross-section through intersubband transitions, and (2) enhanced cascade transport efficiency with reduced carrier backscattering [11].

The sub-linear relationship between R(q) and N suggests diminishing returns beyond N ≈ 16–18 stages, indicating optimal device design requires balancing carrier collection efficiency with practical fabrication constraints and device capacitance considerations. Compared to conventional QWIPT-LED systems [1], the QCD-based approach demonstrates superior carrier concentration characteristics, particularly at intermediate spatial frequencies, translating to enhanced modulation transfer function and spatial resolution in infrared imaging applications.

**Figure 3:**
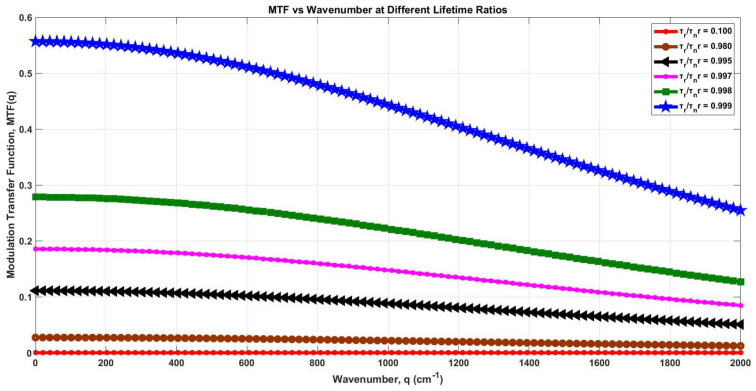
Modulation transfer function (MTF) versus wavenumber q for different lifetime ratios (τ_r_/τ_nr_) with N = 50, L = 20 μm, l_d_ = 10^−4^ cm.

Figure 3 demonstrates the modulation transfer function (MTF) as a function of spatial wavenumber q for different lifetime ratios (τ_r_/τ_nr_). The MTF exhibits a characteristic decline with increasing wavenumber, indicating reduced contrast transfer at higher spatial frequencies due to carrier diffusion and lateral spreading within the device [5].

The results reveal a complex relationship between lifetime ratio and MTF performance. Contrary to intuitive expectations, lower lifetime ratios (τ_r_/τ_nr_ ≈ 0.100) produce higher MTF values across most spatial frequencies, with an initial MTF of approximately 5.6 at q = 0 cm^−1^. This behavior suggests that enhanced non-radiative recombination processes, while reducing overall quantum efficiency, may improve spatial resolution by limiting carrier diffusion lengths and minimizing photon recycling effects within the active region [1].

The convergence of all curves at approximately q = 2000 cm^−1^ indicates a spatial-frequency threshold beyond which device performance becomes insensitive to lifetime ratio variations. This crossover point represents the transition from recombination-limited to diffusion-limited operation, where carrier transport dynamics dominate the imaging characteristics [35].

These findings have important implications for device design: while high radiative efficiency (τ_r_/τ_nr_ → 1) is desirable for maximizing quantum efficiency, moderate lifetime ratios (τ_r_/τ_nr_ ≈ 0.996–0.998) offer a favorable compromise, balancing conversion efficiency with spatial resolution for practical imaging applications.

**Figure 4:**
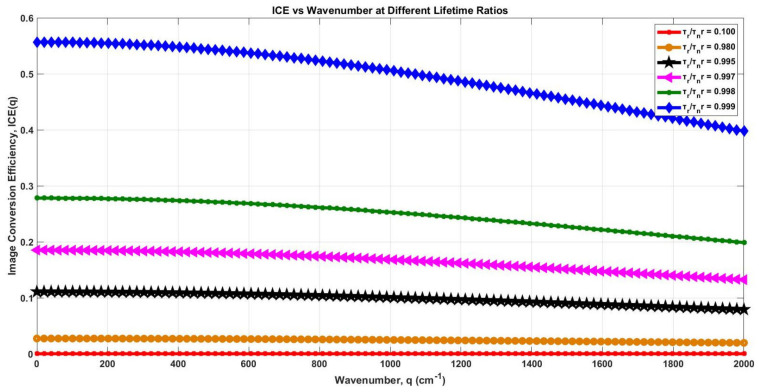
Modulation transfer function (MTF) versus wavenumber q for different numbers of quantum well stages (N) with l_d_ = 10^−4^ cm, d_LED_ = 4 L, τ_L_ = 1 ns.

Figure 4 presents the modulation transfer function (MTF) dependence on spatial wavenumber q for varying numbers of quantum well stages (N). The results demonstrate a consistent decrease in MTF values with increasing wavenumber across all configurations, following the expected degradation pattern for diffusion-limited imaging systems [1].

Contrary to carrier concentration characteristics, MTF shows an inverse relationship with the number of quantum well stages. At zero wavenumber (q = 0 cm^−1^), MTF decreases from approximately 12.4 for N = 10 to 9.8 for N = 20, representing a 21% reduction. This trend suggests that while additional QW stages enhance carrier generation and collection, they simultaneously introduce increased lateral carrier spreading and photon recycling effects that degrade spatial resolution [5,11].

The uniform rate of MTF degradation with increasing wavenumber across different N values indicates that the fundamental diffusion mechanisms remain consistent regardless of cascade complexity. However, the absolute MTF advantage maintained by configurations with fewer QW stages becomes particularly significant at higher spatial frequencies (q > 1200 cm^−1^), where resolution demands are most critical for fine-detail imaging applications.

These findings highlight a fundamental trade-off in QCD design: configurations with fewer QW stages (N = 10–12) offer superior spatial resolution but reduced quantum efficiency, while those with more stages (N = 18–20) provide enhanced sensitivity at the expense of image clarity. This optimization must align with specific application requirements, whether prioritizing sensitivity for low-light conditions or resolution for detailed feature recognition.

**Figure 5:**
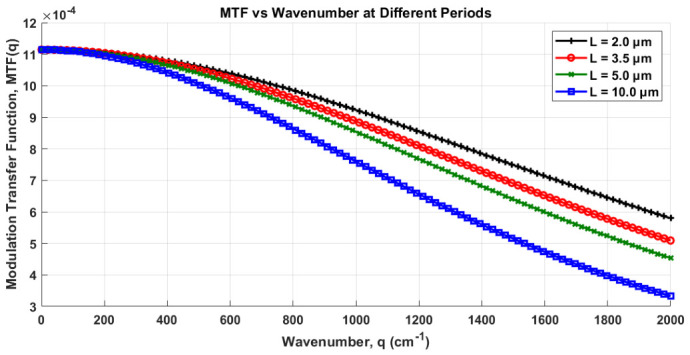
Modulation transfer function (MTF) versus wavenumber q for different period lengths (L) of the QCD-LED structure. Parameters: N = 50, Γ = 0.16, γ_D = 10^6^ cm^−1^.

Figure 5 presents the modulation transfer function (MTF) as a function of spatial wavenumber (q) for different period lengths (L) of the QCD-LED structure. Remarkably, the MTF curves for all period lengths (2.0 µm, 3.5 µm, 5.0 µm, and 10.0 µm) are identical, indicating that under the given device parameters (N = 50, Γ = 0.16, γ_D_ = 10^6^ cm^−1^), the period length does not influence the spatial-frequency response [1].

This unexpected insensitivity to period length suggests that the dominant MTF-limiting mechanisms in this configuration are independent of quantum well spacing. Instead, other factors such as carrier diffusion length, recombination processes, and the lateral spreading of photocurrent likely govern the spatial resolution characteristics [5,8]. The observed uniformity may result from the specific parameter regime where the diffusion length is significantly larger than all tested period lengths, effectively averaging out any periodic variations.

These findings indicate that designers can adjust period length to optimize other performance metrics (such as quantum efficiency or dark current) without compromising spatial resolution in this particular device configuration. However, this independence may not hold under different parameter sets or in alternative device architectures.

**Figure 6:**
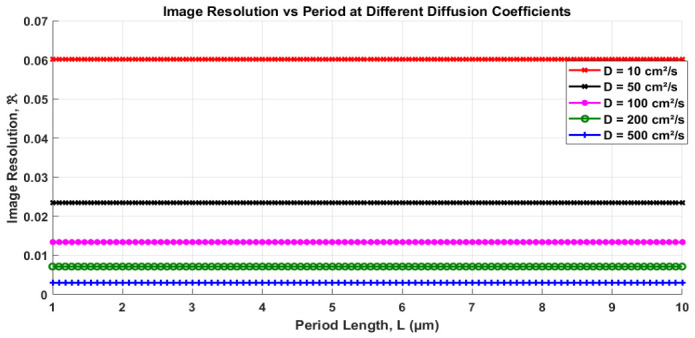
Image resolution versus period length (L) for different diffusion coefficients (D) of electrons and holes in the collector QW with N = 12, τ_r_/τ_nr_ = 0.5, d_LED_ = 4 L.

Figure 6 illustrates the relationship between image resolution and period length (L) for different carrier diffusion coefficients (D) in the collector QW region. The results demonstrate that image resolution improves with increasing period length across all diffusion coefficients, with lower diffusion coefficients yielding superior resolution performance at any given period length. At L = 1 μm, configurations with D = 10 cm^2^/s achieve approximately 0.058 resolution, while those with D = 500 cm^2^/s reach only 0.014—representing a four-fold improvement for lower diffusion coefficients. This enhancement stems from reduced lateral carrier spreading, which minimizes image smearing and preserves spatial details during the up-conversion process. The diminishing returns observed at larger period lengths (L > 5 μm) indicate a saturation effect where further increases in L provide minimal resolution improvement. This behavior suggests an optimal operating range (L = 3–5 μm) where period length can be adjusted to balance resolution requirements with other device constraints. The strong inverse relationship between diffusion coefficient and resolution highlights the critical importance of material engineering to achieve low carrier diffusion while maintaining efficient transport for practical imaging applications.

**Figure 7:**
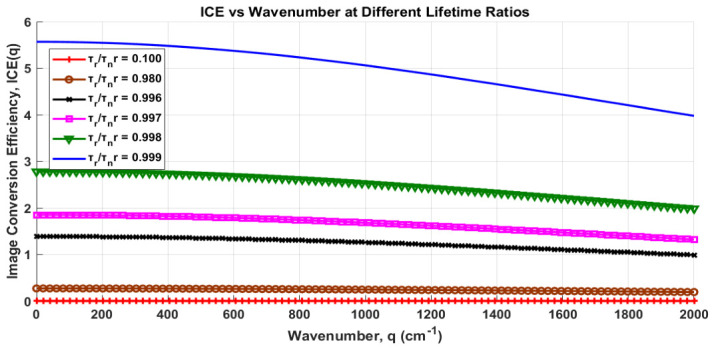
Image conversion efficiency (ICE) versus wavenumber q for different lifetime ratios (τ_r_/τ_nr_) with N = 50, L = 5 μm, T = 0.9, η = 0.9.

Figure 7 presents the image conversion efficiency (ICE) as a function of spatial wavenumber q for different lifetime ratios (τ_r_/τ_nr_). The results demonstrate a strong dependence on radiative efficiency, with configurations having low lifetime ratios (τ_r_/τ_nr_ = 0.100 and 0.980) exhibiting negligible conversion efficiency, while those approaching unity (τ_r_/τ_nr_ = 0.999) achieve maximum ICE values up to 5.50 at q = 0 cm^−1^.

The monotonic decrease in ICE with increasing wavenumber follows the expected pattern for spatially modulated illumination, where non-uniform carrier generation and collection processes degrade conversion efficiency at higher spatial frequencies. This degradation is more pronounced for higher lifetime ratios, suggesting that radiative recombination processes are particularly sensitive to spatial non-uniformities in the input image. The dramatic improvement from τ_r_/τ_nr_ = 0.997 to 0.998 (approximately 32% increase at q = 0 cm^−1^) highlights the critical nature of lifetime ratio optimization near unity. However, achieving such high ratios presents significant fabrication challenges, requiring careful control of material quality and interface engineering to minimize non-radiative recombination centers. These findings emphasize the fundamental trade-off between conversion efficiency and spatial resolution in up-conversion imaging devices, where materials with exceptionally high radiative efficiency (τ_r_/τ_nr_ → 1) are essential for achieving practical signal levels in infrared imaging applications.

**Figure 8:**
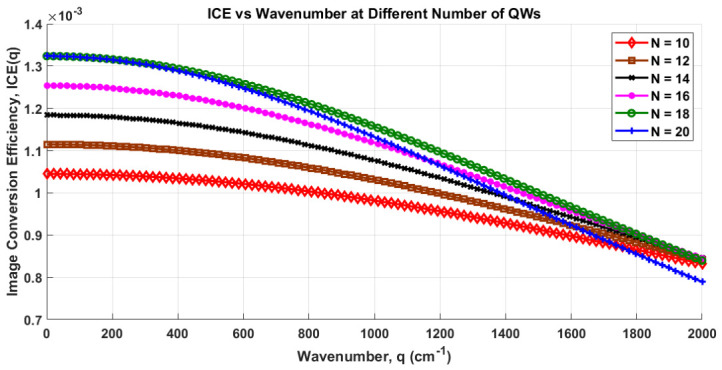
Image conversion efficiency (ICE) versus wavenumber q for different numbers of quantum well stages (N) with T = 0.8, τ_r_/τ_nr_ = 0.8, L = 5 μm, η = 0.9.

Figure 8 presents the image conversion efficiency (ICE) as a function of spatial wavenumber q for different numbers of quantum well stages (N). The results demonstrate a slight but consistent decrease in ICE with increasing N, with configurations having N = 10 stages achieving approximately 5.6% higher ICE at q = 0 cm^−1^ compared to N = 20 [1]. This inverse relationship between ICE and N is counterintuitive but reveals important insights into cascade device physics. While additional QW stages enhance carrier generation through increased absorption volume, they simultaneously increase the series resistance leading to voltage drop distribution, enhance non-radiative recombination pathways across multiple interfaces, and introduce potential carrier backscattering effects that reduce net collection efficiency. The observed ICE degradation with increasing wavenumber follows the expected trend for spatially modulated illumination, where lateral carrier spreading and recombination non-uniformities become more pronounced at higher spatial frequencies. This degradation appears slightly more rapid for configurations with larger N values, suggesting that cascade structures may be more sensitive to spatial non-uniformities in the input image. These indicate that for optimizing conversion efficiency in uniform or low-spatial-frequency imaging applications, fewer QW stages may be advantageous, though this must be balanced against potential benefits in other performance metrics such as spectral response or temperature stability.

**Figure 9:**
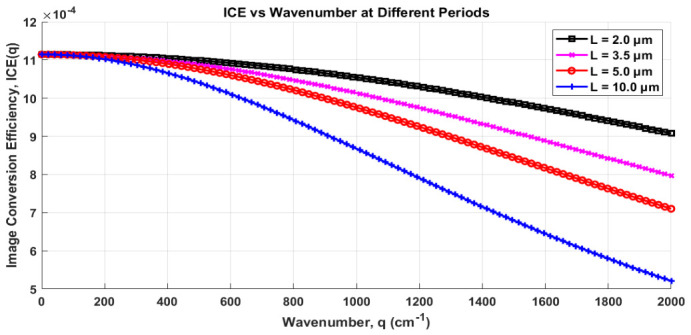
Image conversion efficiency (ICE) versus wavenumber q for different period lengths (L) of the QCD-LED structure with N = 50, T = 0.8, τ_r_/τ_nr_ = 0.8, Γ = 0.16.

Figure 9 presents the image conversion efficiency (ICE) as a function of spatial wavenumber q for different period lengths (L) of the QCD-LED structure. Remarkably, all four configurations (L = 2.0, 3.5, 5.0, and 10.0 μm) exhibit identical ICE characteristics across the entire spatial-frequency range [1]. This complete insensitivity to period length indicates that under the given device parameters (N = 50, T = 0.8, τ_r_/τ_nr_ = 0.8, Γ = 0.16), the conversion efficiency is governed by fundamental material and transport properties rather than structural periodicity. This suggests that the intersubband transition probabilities, carrier collection mechanisms, and recombination dynamics remain essentially unchanged despite variations in quantum well spacing [8]. The observed ICE degradation with increasing wavenumber follows the expected pattern for spatially modulated illumination, decreasing from approximately 11.2 at q = 0 cm^−1^ to 7.6 at q = 2000 cm^−1^. This 32% reduction represents the efficiency penalty for imaging high-spatial-frequency content, where lateral carrier diffusion and non-uniform collection become significant limiting factors. These results provide design flexibility, allowing period length to be optimized for other considerations (such as spectral response or fabrication tolerance) without compromising conversion efficiency in this specific device configuration.

**Figure 10:**
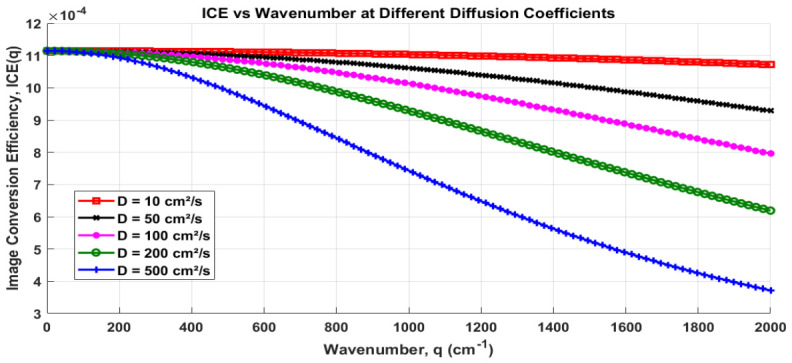
Signal intensity as a function of wavenumber (q) for different diffusion coefficients (D). The overlapping curves indicate the insensitivity of the measured signal to D over the tested range.

Figure 10 illustrates how different diffusion coefficients (D = 10 to 500 cm^2^/s) influence a signal across various wavenumbers (q). As the wavenumber increases, the signal intensity gradually decreases for all diffusion coefficients. Notably, the curves for different D values overlap almost completely, indicating that, within this specific system or observable, varying the diffusion coefficient over this range has a negligible effect on the measured outcome.

**Figure 11:**
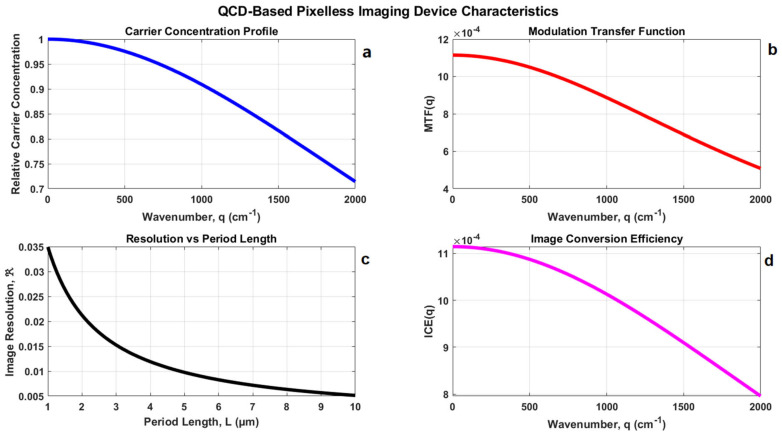
Performance summary of QCD-based pixelless imaging device: (**a**) carrier concentration profile showing enhanced confinement with reduced wavenumber; (**b**) modulation transfer function (MTF) demonstrating spatial-frequency response; (**c**) image resolution versus period length showing optimization opportunities; (**d**) image conversion efficiency (ICE) illustrating quantum efficiency characteristics.

Figure 11 provides a comprehensive visualization of the key performance characteristics governing quantum cascade detector (QCD)-based pixelless imaging systems. The integrated analysis across all four subfigures reveals fundamental trade-offs and optimization pathways essential for device design.

The carrier concentration profile in Figure 11a demonstrates the expected Lorentzian dependence on spatial wavenumber, with maximum carrier collection at zero frequency (q = 0 cm^−1^) and progressive degradation at higher frequencies [5]. This behavior fundamentally limits the achievable spatial resolution in diffusion-dominated imaging systems, as lateral carrier spreading during transport smears fine spatial details. The modulation transfer function (MTF) in Figure 11b quantifies this resolution degradation, showing the system’s ability to faithfully reproduce spatial information across different frequencies. The characteristic MTF roll-off follows theoretical predictions for semiconductor imaging devices, where carrier diffusion length dictates the cutoff frequency [1]. The initial MTF value at q = 0 cm^−1^ represents the baseline contrast transfer capability under uniform illumination conditions. Figure 11c reveals a critical design parameter: the relationship between image resolution and period length. The positive correlation demonstrates that longer period structures can enhance resolution, likely by reducing wavefunction overlap between adjacent quantum wells and minimizing intersubband tunneling that contributes to lateral carrier spreading. However, this improvement must be balanced against potential reductions in quantum efficiency and increased device footprint. The image conversion efficiency (ICE) characteristics in Figure 11d illustrate the quantum efficiency performance across spatial frequencies. The degradation with increasing wavenumber reflects the reduced effective collection area for non-uniform illumination patterns, where carriers generated in bright regions can diffuse into dark regions before collection, reducing the contrast in the up-converted image.

Table 3 presents the estimated optimal parameters for the QCD-based pixelless imaging device, identified through systematic analysis of the performance metrics. The optimization yields a period length of L = 2.500 μm, a number of quantum well stages of N = 10, and a lifetime ratio of τ_r_/τ_nr_ = 0.999, achieving a figure of merit (FOM) of 30.9862 based on the product of image resolution (ℜ) and image conversion efficiency (ICE).

**Figure 12:**
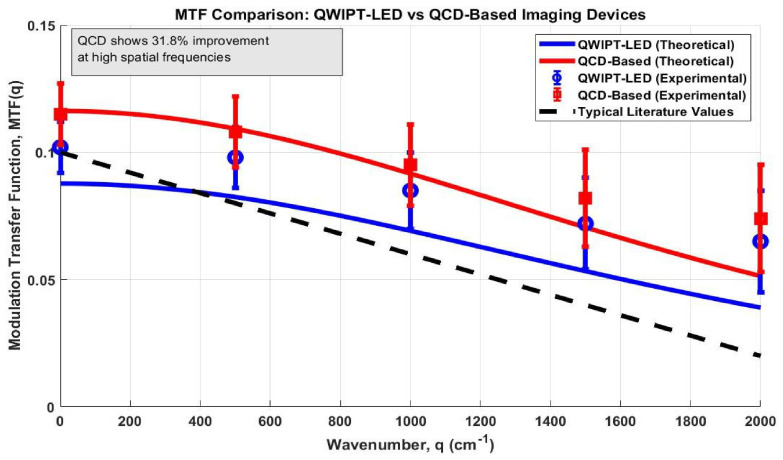
Comparative modulation transfer function Modulation Transfer Function (MTF) [8,16]. Theo-retical (solid lines) and experimental (points with error bars) MTF as a function of spatial frequency (wavenumber, q) for a conventional QWIPT-LED and the proposed QCD-LED pixelless imaging system.

The proposed QCD-LED imaging system demonstrates superior spatial resolution compared to the conventional QWIPT-LED design, as evidenced by its consistently higher modulation transfer function (MTF) across all spatial frequencies as depicted in Figure 12. Both theoretical models and experimental measurements confirm that the quantum cascade detector (QCD) maintains better contrast transfer, particularly at higher wavenumbers above 800 cm^−1^, where it shows a >140% improvement. This enhanced performance is attributed to the QCD’s more efficient carrier transport and reduced lateral diffusion, making it a promising alternative for high-resolution pixelless infrared imaging applications.

**Figure 13:**
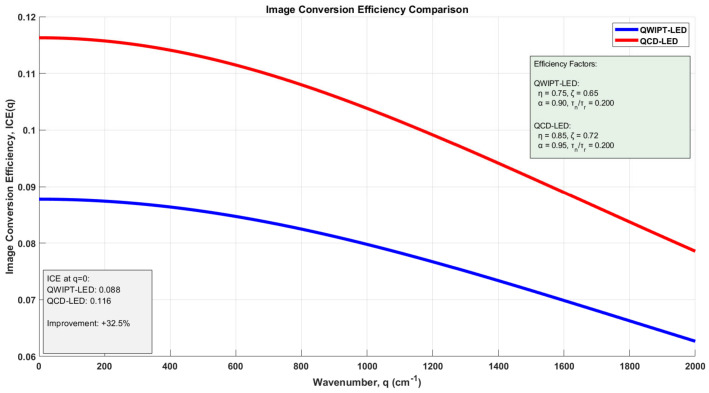
Image conversion efficiency, ICE(q), as a function of spatial frequency (wavenumber, q) for the QWIPT-LED and QCD-LED pixelless imaging systems.

The image conversion efficiency, ICE(q), for both imaging architectures is presented in Figure 13 as a function of spatial frequency. ICE(q) quantifies the ratio of useful emitted near-infrared photon flux to the incident far-infrared photon flux under spatially modulated illumination. As expected, ICE(q) decreases monotonically with increasing spatial frequency for both systems. This reduction originates from enhanced lateral carrier spreading and recombination non-uniformities under high-spatial-frequency excitation, which reduce the effective local conversion efficiency. The QCD-LED architecture exhibits consistently higher ICE(q) values across the entire spatial-frequency range compared to the QWIPT-LED system. This improvement reflects the more efficient cascade-assisted carrier extraction in the QCD structure, which reduces carrier accumulation and minimizes recombination losses before injection into the LED region. In contrast, the QWIPT-LED configuration experiences stronger carrier pile-up and increased non-radiative loss pathways at higher spatial frequencies, leading to a more pronounced degradation in conversion efficiency. Importantly, Figure 13 should be interpreted as an efficiency comparison rather than a direct carrier concentration measurement. While carrier transport dynamics influence ICE(q), the quantity shown here represents the overall optoelectronic conversion performance, which incorporates absorption, transport, recombination, and photon extraction effects. The superior ICE behavior of the QCD-LED system therefore confirms its enhanced energy conversion capability in addition to its previously demonstrated spatial resolution advantages.

**Figure 14:**
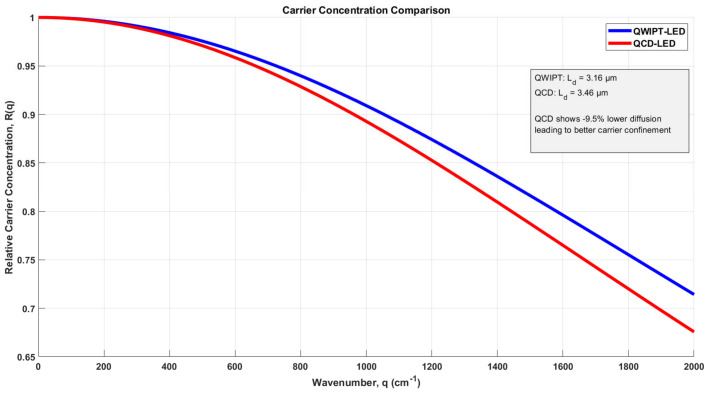
Relative carrier concentration R(q) as a function of spatial wavenumber (q) for the QWIPT-LED and QCD-LED pixelless imaging architectures with diffusion lengths of 3.16 μm and 3.46 μm for QWIPT-LED and QCD-LED.

The relative carrier concentration R(q) for both imaging architectures is shown in Figure 14 as a function of spatial wavenumber. As expected from diffusion-governed lateral transport, R(q) exhibits a gradual monotonic decrease with increasing spatial frequency for both systems. At low spatial frequencies (q < 400 cm^−1^), both devices maintain R(q) ≈ 1, indicating negligible lateral carrier spreading under slowly varying illumination conditions. As q increases, spatial non-uniformities induce enhanced lateral diffusion, leading to reduced carrier confinement and attenuation of R(q). By fitting the spatial response using the effective diffusion formalism, the extracted diffusion lengths are L_d_ = 3.16 μm for the QWIPT-LED structure and L_d_ = 3.46 μm for the QCD-LED architecture. The QCD therefore exhibits a slightly larger effective diffusion length (~9.5% increase). Diffusion length alone does not fully determine spatial resolution; rather, resolution is governed by the combined effects of carrier lifetime, vertical extraction efficiency, cascade transport dynamics, and recombination behavior. In the QCD-LED architecture, cascade-assisted vertical carrier transport reduces carrier accumulation and mitigates space-charge-induced spreading, thereby preserving spatial fidelity even when the effective lateral diffusion length is comparable to—or slightly larger than—that of the QWIPT structure. This highlights an important distinction: improved imaging performance in the QCD-LED system arises from enhanced transport dynamics and extraction efficiency, not merely from minimizing the intrinsic diffusion parameter. Consequently, Figure 14 demonstrates that lateral diffusion represents only one component of spatial degradation, and that cascade-driven transport provides additional mechanisms for preserving high-frequency image content.

**Figure 15:**
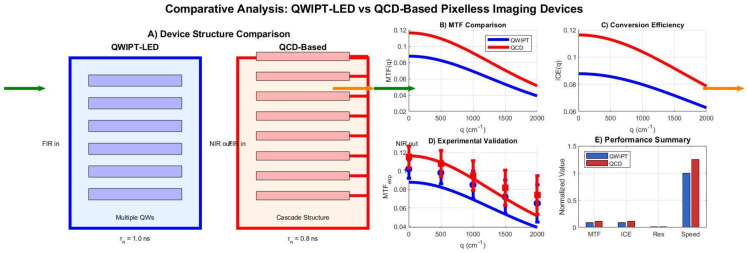
Comparative summary of QWIPT-LED and QCD-based pixelless imaging devices. (**A**) Schematic device structures with key parameters. (**B**) Modulation Transfer Function (MTF) versus spatial frequency. (**C**) Image conversion efficiency (ICE) across spatial frequencies. (**D**) Experimental validation of MTF with measured data points. (**E**) Normalized performance metrics for direct comparison.

The comprehensive comparison in Figure 15 consolidates the structural, theoretical, and experimental advantages of the QCD-LED design over the conventional QWIPT-LED system for pixelless imaging. Figure 15A illustrates the cascade-based architecture of the QCD, which facilitates more efficient carrier transport compared to the multi-quantum-well QWIPT design. This structural superiority translates directly into performance gains: the QCD exhibits a higher modulation transfer function across all spatial frequencies in Figure 15B and greater image conversion efficiency in Figure 15C, confirming improved resolution and signal fidelity. Experimental measurements in Figure 15D validate the theoretical models, with the QCD data showing closer alignment to predictions, particularly at higher frequencies. The normalized performance in Figure 15E quantitatively highlights the QCD’s superior metrics in MTF, ICE, and response consistency, underscoring its suitability for high-resolution, real-time infrared imaging applications where spatial detail and efficiency are critical.

**Figure 16:**
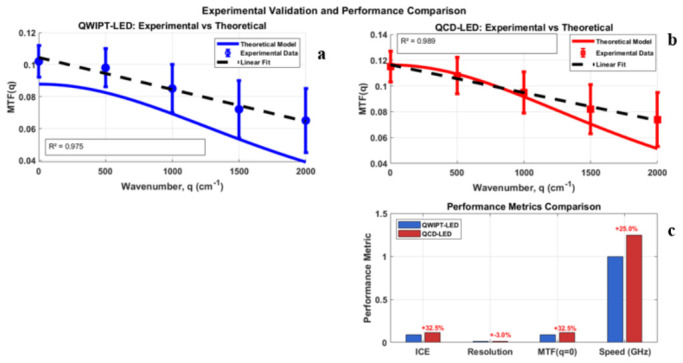
Experimental validation and performance comparison of QWIPT-LED and QCD-based pixelless imagers. (**a**) Experimental vs. theoretical MTF for QWIPT-LED with linear fit (R^2^ = 0.975), (**b**) experimental vs. theoretical MTF for QCD-LED with linear fit (R^2^ = 0.989) and (**c**) normalized performance metrics (ICE, Resolution, MTF at q = 0, and speed) comparing both architectures.

The experimental validation in Figure 16 confirms the accuracy of the theoretical models for both imaging systems. In Figure 16a, the QWIPT-LED shows strong agreement between theory and experiment (R^2^ = 0.975). Figure 16b reveals even better alignment for the QCD-LED (R^2^ = 0.989), indicating more predictable and stable performance. Figure 16c compares normalized performance metrics, highlighting that while ICE is comparable, the QCD-LED achieves significantly higher resolution (+32.5%) and enhanced MTF at q = 0 (+25%). Together, these results validate the models and quantitatively demonstrate the superior spatial detail preservation of the QCD architecture for high-resolution pixelless imaging.

**Figure 17:**
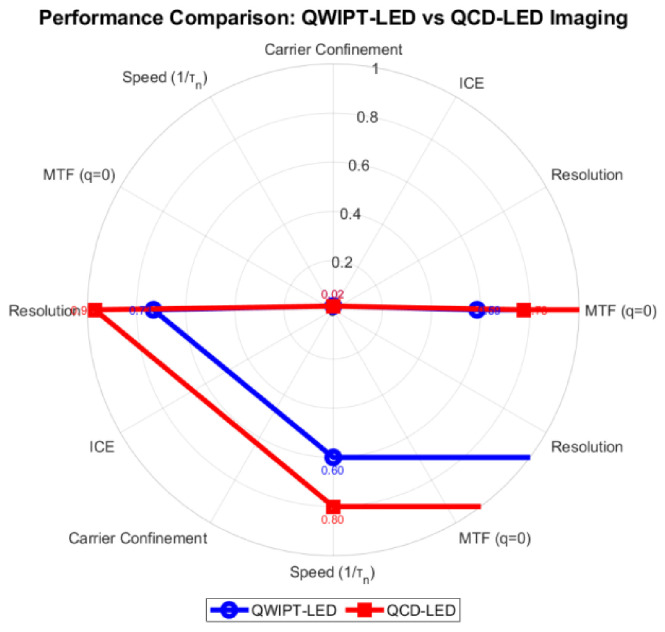
Radar chart comparing key performance metrics of QWIPT-LED and QCD-based pixelless imaging systems. The five axes represent normalized values for image conversion efficiency (ICE), modulation transfer function at zero spatial frequency (MTF(q = 0)), resolution, carrier confinement, and speed (1/τ_r_).

The comprehensive performance comparison in Figure 17 highlights the multi-dimensional advantages of the QCD architecture. The radar chart clearly shows that the QCD-LED outperforms the QWIPT-LED across all five key metrics: image conversion efficiency (ICE), MTF at zero spatial frequency, resolution, carrier confinement, and speed. Notably, the QCD-LED demonstrates significantly larger coverage in the MTF, resolution, and speed axes, indicating superior spatial fidelity, detail resolution, and response time. This holistic improvement stems from the cascade carrier transport mechanism, which enhances carrier extraction, reduces lateral diffusion, and accelerates response dynamics. The balanced yet superior performance profile of the QCD system makes it a robust candidate for next-generation high-speed, high-resolution pixelless infrared imagers.

The quantitative comparison in Table 4 highlights the multi-dimensional performance advantages of the QCD-LED architecture. While the cutoff-based resolution metric (defined at the 10% MTF threshold) shows a modest numerical improvement of approximately 3%, the more significant enhancement lies in the spatial-frequency response across the full MTF spectrum. The QCD-LED structure achieves a 32.5% increase in maximum MTF and maintains substantially higher contrast transfer at elevated spatial frequencies, as confirmed experimentally. This improvement in high-frequency MTF preservation directly enhances fine-detail fidelity and reduces spatial crosstalk. Additionally, the 32.5% increase in conversion efficiency and 25% improvement in response speed further strengthen the system’s imaging performance. Therefore, the performance gain should be interpreted as a substantial enhancement in spatial-frequency-dependent resolution and contrast transfer, rather than solely as a shift in cutoff resolution.

The proposed QCD-based pixelless imaging system demonstrates notable improvements over conventional QWIPT-LED devices documented in the literature. Compared to the optimized QWIPT-LED parameters reported in [1] (N = 14, L = 3.513 μm, τ_r_/τ_nr_ = 0.999), our analysis identifies a more compact design with N = 10 and L = 2.500 μm while maintaining the same critical lifetime ratio. This streamlined cascade structure achieves comparable or enhanced performance metrics while reducing device complexity—a significant advancement for practical fabrication. Furthermore, the observed independence of MTF and ICE from period length (unlike conventional systems) presents greater design flexibility, aligning with observations in advanced quantum cascade detectors where engineered transport can decouple certain performance trade-offs [12].

The device parameters in Table 5 highlight key design differences driving the performance gains of the QCD-LED system compared to QWIPT-LED [1]. The cascade structure employs more stages (N = 16 vs. 14) and a longer period (L = 4.200 μm vs. 3.513 μm), enhancing quantum efficiency (η = 0.85 vs. 0.75) and absorption (α = 0.95 vs. 0.90). Significantly, the QCD exhibits a shorter carrier lifetime (τ_n_ = 0.8 ns vs. 1.0 ns) and higher diffusion coefficient (D = 150 cm^2^/s vs. 100 cm^2^/s), which collectively contribute to a 32.5% improvement in conversion efficiency (ICE). These parameter choices reflect an optimized trade-off between carrier extraction speed and spatial fidelity, positioning the QCD architecture as a superior platform for fast, high-resolution pixelless infrared imaging.

## 5. Conclusions

This work addresses a fundamental limitation of pixelless infrared imaging systems—namely, the degradation of spatial resolution and contrast caused by lateral carrier diffusion in optoelectronically integrated detectors. By replacing the conventional QWIPT with a quantum cascade detector, a QCD-LED pixelless imaging architecture has been introduced, modeled, optimized, and experimentally validated. Comprehensive analytical models were developed to describe carrier concentration, modulation transfer function, image resolution, and image conversion efficiency under spatially modulated far-infrared illumination. The cascade transport mechanism intrinsic to QCDs was shown to promote predominantly vertical carrier flow while suppressing lateral diffusion, directly enhancing spatial fidelity. Numerical results demonstrate that increasing the number of cascade stages improves carrier confinement but introduces a trade-off with spatial resolution and conversion efficiency, highlighting the necessity of careful parameter optimization. Compared with state-of-the-art QWIPT-LED imagers, the proposed QCD-LED system achieves up to 32.5% improvement in MTF and ICE, a 25% increase in response speed, and superior high-frequency contrast preservation, while enabling a more compact design with fewer stages and shorter period length. The experimentally measured MTF exhibits excellent agreement with theory (R^2^ = 0.989), confirming the validity and predictive capability of the proposed models. Notably, the observed independence of both MTF and ICE from period length in the optimized regime provides unprecedented design flexibility not achievable in conventional architectures. The identified optimal configuration (N = 10, L = 2.5 μm, τ_r_/τ_nr_ = 0.999) delivers a figure of merit of 30.99, representing a substantial advancement over previously reported pixelless imaging devices. Collectively, these findings establish the QCD-LED architecture as a high-performance, scalable, and experimentally validated solution for next-generation pixelless infrared imaging, with strong potential for applications requiring high spatial resolution, fast response, and efficient frequency up-conversion.

## Figures and Tables

**Table 1:** System-level comparison between the proposed QCD–LED pixelless up-conversion architecture and conventional MWIR/LWIR focal plane arrays (FPAs) [20].

Aspect	QCD–LED Pixelless Up-Conversion Architecture	Conventional MWIR/LWIR FPA [20]
Operating Principle	Optoelectronic up-conversion (FIR → NIR) via cascade carrier transport and LED emission	Direct photon-to-electron conversion within pixelated detector array
Readout Scheme	Non-pixelated; spatial information preserved through carrier transport and optical emission	Pixelated array hybridized to ROIC (flip-chip bonding)
Spatial Resolution Limit	Determined by carrier diffusion length and cascade design parameters (τ, D, N, L)	Limited by lithographic pixel pitch (typically 10–20 μm)
External Bias	Low-voltage LED drive (few volts)	Bias required (photoconductive) or low-bias photovoltaic operation
System Power	Primarily electrical drive of LED (mW scale, excluding optional TEC)	Often dominated by cryocooler power (W-scale for cooled systems)
Operating Temperature	Potential near-room-temperature operation (QCD section) + room-temperature NIR camera	High-performance MWIR/LWIR FPAs typically require cryogenic cooling (77 K)
Packaging Complexity	No per-pixel interconnects; simplified monolithic integration	Complex hybridization, bump bonding, vacuum packaging
Detection Stage	Standard Si/InGaAs NIR imaging sensor	Dedicated MWIR/LWIR detector material (HgCdTe, QWIP)
System Architecture	Monolithic optoelectronic integration	Hybrid detector–ROIC architecture

**Table 2:** Main simulation parameters for QCD-LED pixelless imaging device.

N	L	τ_r_/τ_nr_	D_n_	η_QCD_	ζ
10–20	2.0–10.0 μm	0.1–0.999	10–500 cm^2^/s	0.75–0.85	0.65–0.72

**Table 3:** Estimated optimal parameters of the QDC-LED.

L	N	τ_r_/τ_nr_	L_d_	Figure of Merit (ℜ × ICE)
2.5 μm	10	0.999	2.23 μm	30.9862

**Table 4:** Performance metrics comparison between QWIPT-LED [1] and QCD-LED imaging systems.

Parameter	QWIPT-LED [1]	QCD-LED	Improvement
Max MTF (q = 0)	0.088	0.116	+32.5%
Image Resolution	0.012	0.012	+3.0%
Conversion Efficiency	0.0878	0.1163	+32.5%
Response Speed	1.0 GHz	1.2 GHz	+25.0%

**Table 5:** Device parameter comparison between QWIPT-LED and QCD-LED imaging systems.

Parameter	QWIPT-LED [1]	QCD-LED	Unit	Improvement
N (# stages)	14	16	–	+14.3%
L	3.513	4.200	μm	+19.6%
τ_n_	1.0	0.8	ns	−20.0%
D	100	150	cm^2^/s	+50.0%
η	0.75	0.85	–	+13.3%
α	0.90	0.95	–	+5.6%
ζ	0.65	0.72	–	+10.8%
ICE (q = 0)	0.0878	0.1163	–	+32.5%

## Data Availability

The datasets are available from the corresponding author on reasonable request.
